# Does Short-Distance Migration Facilitate the Recovery of Black-Necked Crane Populations?

**DOI:** 10.3390/ani15152304

**Published:** 2025-08-06

**Authors:** Le Yang, Lei Xu, Waner Liang, Jia Guo, Yongbing Yang, Cai Lyu, Shengling Zhou, Qing Zeng, Yifei Jia, Guangchun Lei

**Affiliations:** 1 School of Ecology and Nature Conservation, Beijing Forestry University, Beijing 100083, China; yangle@bjfu.edu.cn (L.Y.); ecoxulei@163.com (L.X.); vandy0303@outlook.com (W.L.); guojia.eco@foxmail.com (J.G.); lucai.wetland@foxmail.com (C.L.); zengqing@bjfu.edu.cn (Q.Z.); 2 Key Laboratory of Biological Resources and Biosafety, Institute of Plateau Biology Research of Xizang Autonomous Region, Lhasa 850000, China; 18270292589@139.com (Y.Y.); zsl13518916139@163.com (S.Z.); 3 Center for East Asian-Australasian Flyway Studies, Beijing Forestry University, Beijing 100083, China

**Keywords:** *Grus nigricollis*, migration strategy, satellite tracking, habitat use, Tibetan Plateau, Central Asian Flyway

## Abstract

Understanding the migratory strategies of plateau-endemic species is essential for informing effective conservation, especially under climate change. The Black-necked Crane (*Grus nigricollis*), a high-altitude specialist, has shown notable population growth in recent years. We analysed satellite tracking data from 16 individuals of a western subpopulation in the lake basin region of northern Tibet (2021–2024), focusing on migration patterns, stopover use, and habitat selection. This subpopulation exhibited short-distance (mean: 284.21 km), intra-Tibet migrations with low reliance on stopover sites. Autumn migration was shorter, more direct, higher in altitude, and slower in speed than spring migration. Juveniles used smaller, more fragmented habitats than subadults, and their spatial range expanded over time. Given these patterns, we infer that the short-distance migration strategy may reduce energetic demands and mortality risks while increasing route flexibility—characteristics that may benefit population growth. We refer to this as a low-energy, high-efficiency migration strategy, which we hypothesise could support faster population growth and enhance resilience to environmental change. We recommend prioritizing the conservation of short-distance migration corridors, such as the typical lake basin area in northern Tibet–Yarlung Tsangpo River system, which may help sustain plateau-endemic migratory populations under future climate scenarios.

## Simple Summary

Black-necked Cranes are high-altitude birds that only grow and breed on plateaus. Understanding how they migrate is important for their conservation. In this study, we tracked 16 cranes from northern Tibet using satellite tags. We found that these cranes presented shorter migration distances and were less reliant on stopover sites compared to other subpopulations. The autumn migration was shorter, higher in altitude, and slower in speed, while spring migration was longer and more complex. Younger cranes used smaller and more scattered habitats, which gradually expanded as they grew older. We deduce that short-distance migration may help cranes conserve energy, decrease mortality rate during migration, and allow more flexibility in migration routes. This could be a driver for the continued growth of this subpopulation, which could support population growth in the greater region by spillover and inter-population communications. Protecting key habitats along their short routes is important for conserving these birds, especially as the environment continues to change due to climate impacts.

## 1. Introduction

Migration dynamics, wintering and breeding ecology, and stopover site usage are important aspects of migratory waterbird studies, and are crucial for the conservation of migratory waterbird species and their habitat networks [1]. Migration in waterbirds, similar to other migratory taxa, is a strategy for tracking food resources to better conduct activities as breeding and wintering, as well as to mitigate competition and avoid predation. Therefore, it has long been a core topic for conservationists on how to manage the threats to the migratory routes of a waterbird species, and coordinate all stakeholders to conduct conservation actions [2]. Waterbirds are sensitive to environmental change, and have been considered as bio-indicators for ecosystem health and conservation effectiveness [3]. Among the nine flyways around the world, the Central Asian Flyway is the least studied flyway, even though it is one of the most threatened, with 40% of its species in decline [4]. Albeit being the shortest, it spans across a large range of countries and a variety of terrains. Long-term, large-scale studies on migratory species on this flyway are scarce on this flyway, and there’s a lack of research on the population trends and migratory strategies of its species.

The scattered wetlands on the Tibetan Plateau give it the title of Asian Water Tower. It supports migratory waterbirds with its chains of wetlands as roosting and foraging sites [5] and is a key component of the Central Asian Flyway. However, due to its high altitude of 4000 m, it is one of the most impacted regions under global warming, challenging bird survival through its extreme cold, low humidity and reduced air density [6,7,8]. Waterbird species have adapted unique migratory strategies tailored to this environment [9]. Studies in this field have been concentrated on the more famous migrations, including the flight over the Himalaya mountains [10]. In contrast, studies conducted on the species endemic to the Tibetan Plateau were scarce.

Black-necked Cranes (*Grus nigricollis*) are the only crane species that spends its entire life history on plateaus out of the total of 15 crane species in the world. It is also the last crane species to be discovered [11]. It is a first-class protected animal in China, and is classified as Near Threatened by the International Union for Conservation of Nature (IUCN) Redlist [12]. The Black-necked Crane consists of three populations, namely the western population, the central population and the eastern population. The 10,000-individual Western population in Tibet is the largest. Black-necked Crane is considered an important eco-indicator and a flagship species for the alpine wetland ecosystem [13]. The species is ideal for studying the migratory strategy of birds at high altitudes. So far, past research has discovered 14 unique migratory routes for the Black-neck Crane among its three populations [14,15,16,17,18,19,20]. Those studies provide insights into the long-distance migration on the Qinghai-Tibetan Plateau. However, the migration of the Black-necked Cranes of the western population is still limited, especially the breeding subpopulation of the northern Tibetan lake basin (NT), which has been increasing rapidly (Yang, unpublished data). To resolve this problem, this research used satellite tracking to explore the migration route of Black-necked Cranes in the NT subpopulation. We aim to: (1) Identify the migration route of the subpopulation, (2) analyse the patterns of this migration route between spring and autumn migration, and between juveniles and subadults.

## 2. Materials and Methods

### 2.1. The NT Subpopulation

**Figure 1:**
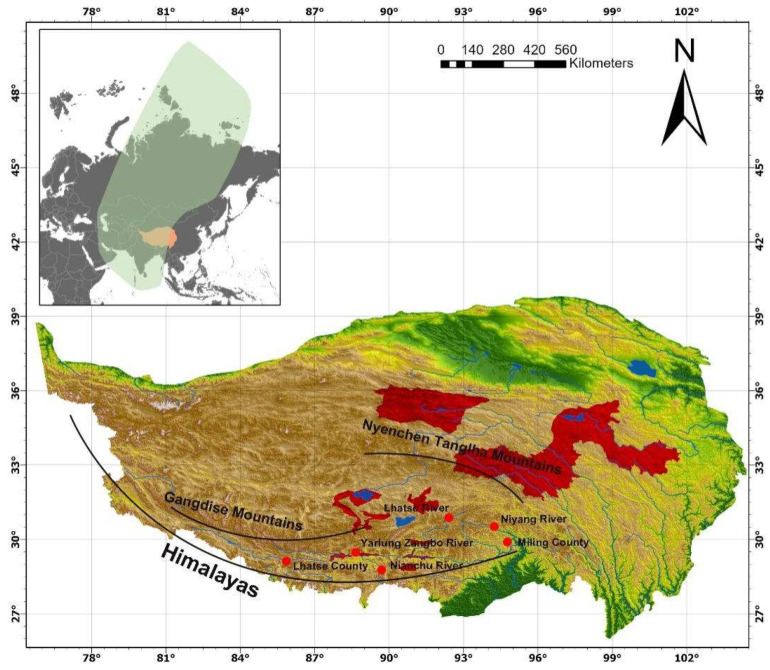
Map of Tibet. The plot on the top left shows the location of Tibet (orange) in relation to the Central Asian Flyway (green). The main map shows the major mountains (annotation) and protected areas (red) in Tibet.

In this study, we focused on the NT breeding subpopulation of the Black-necked Crane. The NT subpopulation is a part of the Western population. Previous studies [21,22] have shown that the NT subpopulation breeds in Selin Co National Reserve (SLCR), which includes Selin Co Lake, Shenzha River, Pengco Lake, among others, with an average altitude of the 4500 m; it migrates the Black-necked Crane National Nature Reserve on the Middle Reaches of the Yarlung Tsangpo River in Tibet Autonomous Region (YZMR) in Southern Tibet, with an average altitude of 3500 m. The subpopulation mostly spends all its life stages inside Tibet (Figure 1); however, migrants from and to the breeding communities of Hoh Xil National Nature Reserve (HXR) (Western Qinghai, with an average altitude of 4600 m); Sanjiangyuan National Nature Reserve (SJYR) (Southern Qinghai, with an average altitude of 4000–4800 m) has been recorded.

Breeding surveys during 2021–2024 showed an increase in the subpopulation. Breeding surveys covered known breeding locations in the NT from past records or reported by local communities. Some known locations were not included in the survey due to logistical issues. In 2021, 59 breeding pairs were found; in 2022, 62 breeding pairs, and in 2023, 75 breeding pairs.

### 2.2. Bird Capturing and Satellite Tracking

We captured and satellite tracked a total of 16 Black-necked Cranes in SLCR during the breeding season of 2021 and 2023. Fifteen of them were flightless juveniles, and one was a moulting adult. Satellite trackers (HQLG4037S, Hunan Global Messenger Technology Co., Ltd. (Changsha, China), weighing 37~44 g) were attached to the right upper leg of the individuals. Birds were weighted and made sure the satellite trackers weighed less than 3% of each bird’s body mass to minimise the impact on its behaviour [23,24]. Location, speed, direction, altitude, and temperature data were transmitted through the GSM system every hour or every three hours, depending on battery efficiency. Bird capture, tracking, and tagging were conducted under the permits of the National Forestry and Grassland Administration, China (Permit No. Lin Hu Xu Zhun (2021) 223; issued on 31 December 2021).

The satellite data accuracy was classified into 5 levels: levels A (error within 5 m), B (5–10 m), C (10–20 m), D (20–100 m), E (>100 m). We only used data with an accuracy level of C and above to ensure credibility. From 2021 to 2024, we collected 147,782 location data points from the 16 tracked individuals (Table 1).

### 2.3. Routes and Seasonal Differences in Migration

Two stages were identified from the tracking data: staging or flying. Staging data was characterised with a speed of 0, and the flying data was characterised with a speed faster than 10 km/h. Using the classified data, through the Geometric function in ArcGIS Pro 3.5.1, we constructed the migration route of the tracked Black-necked Crane individuals. Among the individuals, only one died before completing a migration cycle; all the others migrated at least one cycle. This resulted in 16 complete spring migrations and 18 complete autumn migrations.

We also calculated the migration distance (accumulated distance), migration straightness (shortest distance between start and end divided by accumulated distance), migration speed (accumulated distance/total migration time), flight height (flight altitude subtracted by corresponding terrain elevation) for each migration event [16,25]. The start and end of a migration event were defined as the time when an individual arrived at/departed from a breeding/summering/wintering ground.

We identified stopover sites used in each migration event. A stopover site was defined as a location during a migration event where the individual stayed for more than six hours within a 10 km^2^ area. The stopover duration was calculated for each site. A stopover does not necessarily indicate a refuelling site; it may also serve other purposes such as resting or waiting for favourable conditions.

Statistical tests were performed on the above parameters between spring and autumn migration. For data with a normal distribution (*p* > 0.05 in the Shapiro–Wilk test), we performed *t*-tests. Conversely, for data without a normal distribution (*p* < 0.05 in the Shapiro–Wilk test), we performed Wilcoxon rank-sum tests. All statistical analyses were conducted in R version 4.2.2 [26], using the stats package [27], which is included in the base R distribution.

### 2.4. Analysis of Habitat Utilization and Distribution

We used the Dynamic Brownian Bridge Movement Models (DBBMM) from the move package [28] of R to construct the utility distribution (UD) of each migration event. UD uses percentages to describe the probability distribution of an animal’s spatial use, i.e., the probability of presence of said animal in a certain location. We used 50% and 95% UD in our study to represent the habitat use for each migration event. The areas of 50% UD and 95% UD were calculated. The habitat area (95% UD) was compared between juvenile and subadult, and a Wilcoxon rank-sum test was conducted.

## 3. Results

### 3.1. Migration Route and Seasonal Variation

Most individuals (*n* = 12) showed a consistent migration route between years. The migration of the NT subpopulation was characterised by its short distance, short duration and low dependency on stopover sites and high inter-individual variations.

**Figure 2:**
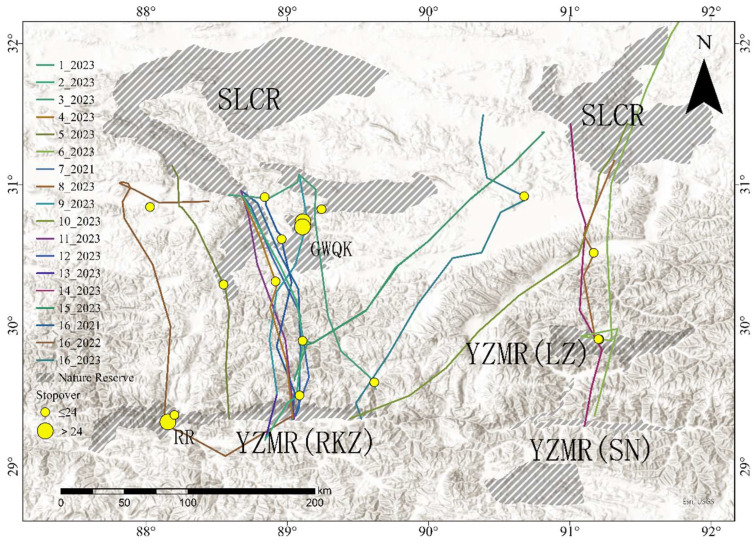
Autumn migration routes and stopover sites. The Black-necked Crane National Nature Reserve on the middle reaches of the Yarlung Tsangpo River in Tibet Autonomous Region (YZMR), Selin Co National Nature Reserve (SLCR), Re River (RR), GeWangQueKang (GWQK).

During autumn migration (Figure 2), stopover sites were used in 16 out of 18 migration events. In total, 16 stopover sites were identified for autumn migration, with an average of 0.89 ± 0.58 stopover sites used per autumn migration event. The stopover duration per site was an average of 1.69 ± 1.87 days. All autumn migrations were conducted within Tibet, starting from the wetlands in SLCR, and ending at the wintering site of YZMR. Three major routes can be concluded between the individuals: ① West of SLCR (Shenzha river, Geren lake, the river of Mujiu, etc.) -YZMR (Rikaze, RKZ) (*n* = 11); ② East of SLCR (Peng Lake, Coer lake, Cuona Lake, etc.) -YZMR (Linzho, LZ/Shannan, SN) (*n* = 3); ③ East of SLCR-YZMR (RKZ) (*n* = 4).

**Figure 3:**
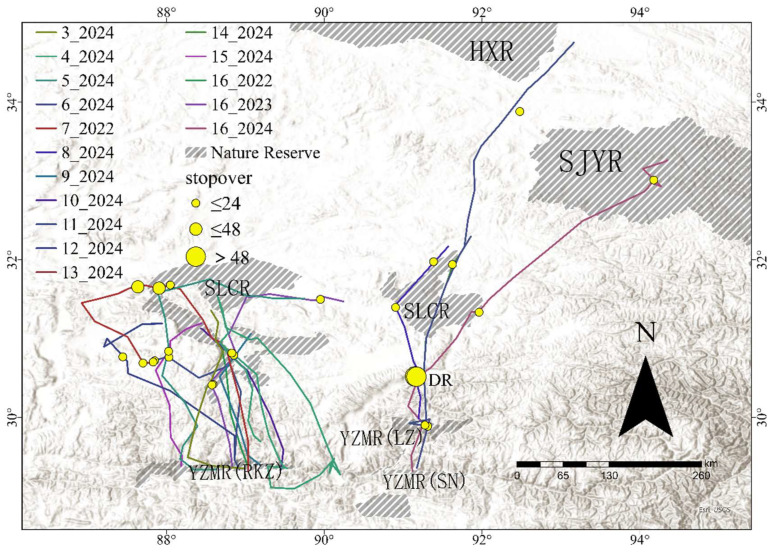
Spring migration routes and stopover sites. The Black-necked Crane National Nature Reserve on the middle reaches of the Yarlung Tsangpo River in Tibet Autonomous Region (YZMR), Selin Co National Nature Reserve (SLCR), Dang River (DR), Hoh Xil National Nature Reserve (HXR), Sanjiangyuan National Nature Reserve (SJYR).

During spring migration (Figure 3), stopover sites were used in 12 out of 16 migration events. In total, 13 stopover sites were identified for spring migration, with an average of 2.16 ± 0.93 stopover sites used per spring migration event. The stopover duration per site was an average of 3.28 ± 7.04 days. 14 spring migrations were conducted within Tibet. Two migration events were conducted across both Tibet and Qinghai. The spring migration routes within Tibet were similar to the autumn migration routes described above, while the two involving Qinghai were: ① YZMR (SN)-HXR (the Beilu River) (*n* = 1); ② YZMR (SN)-SJYR (Zhana River) (*n* = 1).

**Figure 4:**
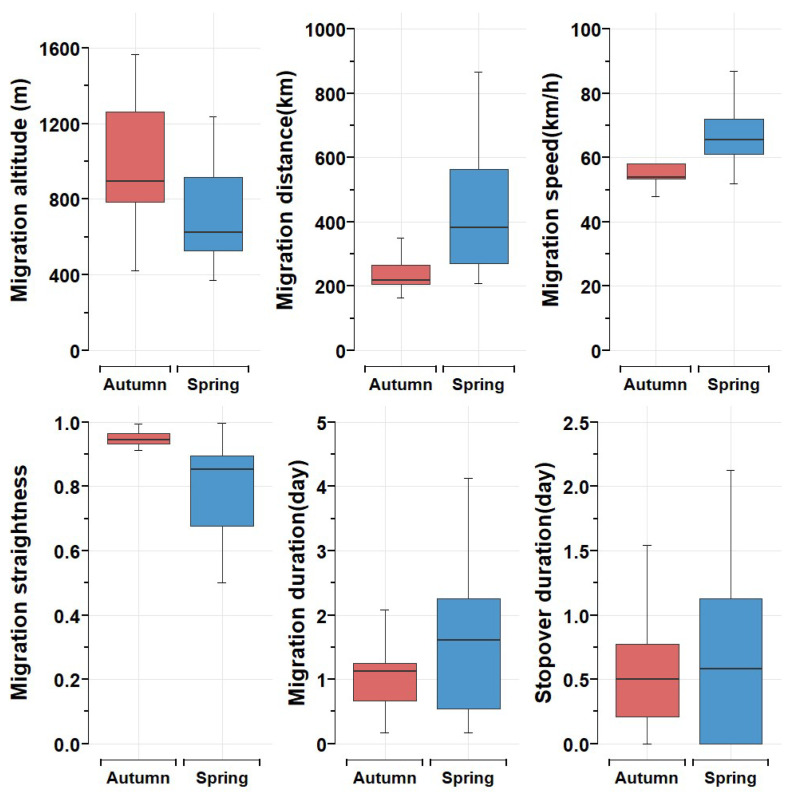
Migration comparison between autumn and spring migration.

The autumn migration was conducted from 18 October to 6 November. The spring migration was conducted from 22 March to 25 May. Comparison between autumn and spring migration (Figure 4) showed significant difference in migration distance (autumn: 235.58 ± 45.87 km; spring: 428.70 ± 188.42 km; *p* = 0.0001), migration altitude (autumn: 979.74 ± 362.03 m; spring: 696.73 ± 248.07 m; *p* = 0.012), migration speed (autumn: 56.64 ± 7.91 km/h; spring: 181.8 ± 93.2 km/h; *p* = 0.002), migration straightness (autumn: 0.89 ± 0.13; spring: 0.75 ± 0.21; *p* = 0.002). There was no significant distance in migration difference (autumn: 1.69 ± 1.67 days; spring: 4.15 ± 7 days; *p* = 0.27) and stopover duration (see above, *p* = 0.66) between the two seasons. Overall, the spring migration was longer in distance, faster in-flight speed, the route was less straight and direct, and flight was lower.

Among the identified stopover sites, the majority were utilized by only a single individual, typically located in small and fragmented wetlands such as tributaries or sub-lakes. In contrast, a few sites were used by multiple individuals, either during different migration seasons or across different years. For example, the GeWangQueKang was used exclusively by individuals 9 and 13 during separate autumn migrations. The Dangqu River was visited by individuals 8 and 16, but in different years. Linzhou county, located near the wintering grounds, functioned as a key corridor node and was used repeatedly: by individuals 6 and 14 during autumn migration in 2023, and again by individual 6 during spring migration in 2024. Notably, simultaneous use of Linzhou was observed, with individuals 6 and 14 occupying the site during overlapping migration periods across both spring and autumn seasons.

### 3.2. Habitat Use

**Figure 5:**
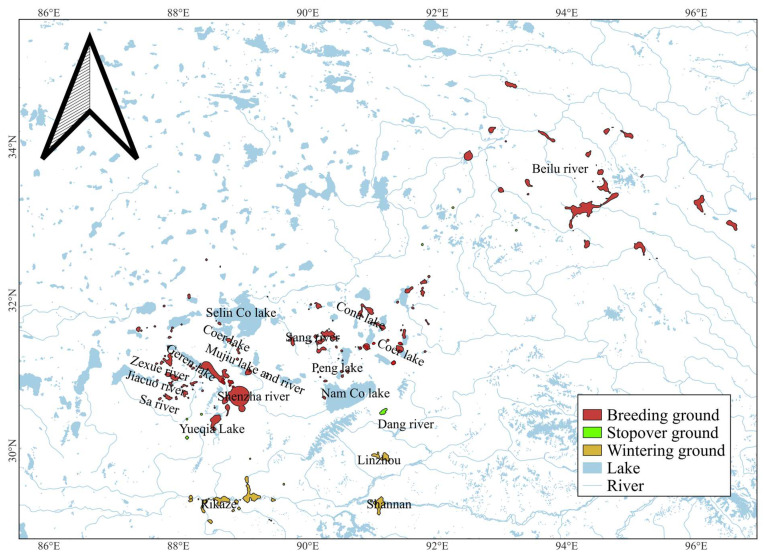
Habitat used by Black-necked Cranes during migration.

Our results from the DBBMM models identified 252 habitats used (Figure 5). The total habitat area (95% UD) was 6773.99 square kilometres, and the core area used (50% UD) comprised 131.6 square kilometres. For wintering grounds, the total area was 2077.80 square kilometres, and the core area was 82.5 square kilometres. The wintering grounds were mainly concentrated at the middle reaches of the Yarlung Zangbo River valley (Rikaze area and Shannan City’s Jiedexiu Forest National Park) and at the Kazi Reservoir and Hutoushan Reservoir in Linzhou County, Lhasa City. For breeding grounds, the total area was 49,696.18 square kilometres, and the core area was 47.27 square kilometres. The breeding area includes sub-lakes and rivers in northern Tibet, such as Shenzha River–Geren Lake, Coer Lake, Mujiu Lake and river, Sang River, Peng Lake–Coer Lake, Yueqia Lake and Beilu River, etc. Stopover sites have a total area (95% UD) of 111.32 square kilometres, including the Sa River, Nam Co Lake–Dang River, and Zexue River–Jiacuo River.

**Figure 6:**
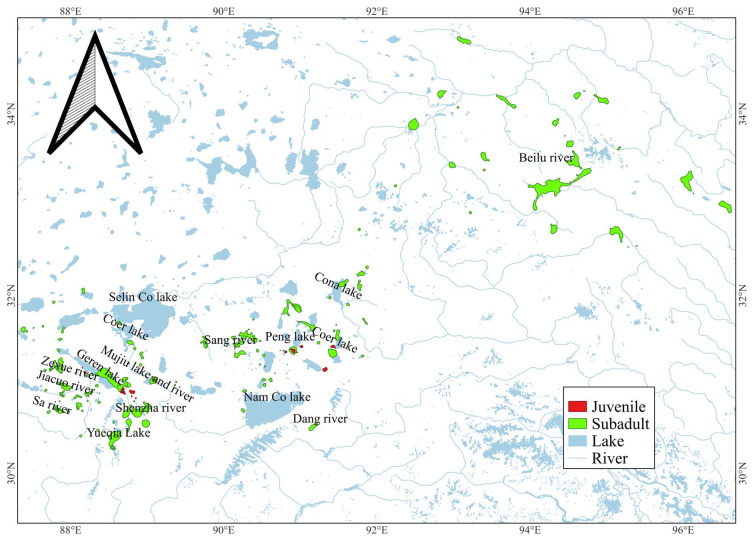
Habitat used by juvenile and subadult Black-necked Cranes.

**Figure 7:**
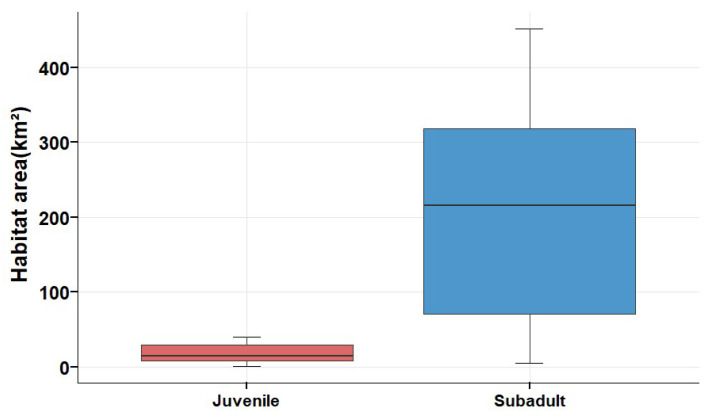
Comparison of habitat area utilised by black-necked cranes by each juvenile or subadult per year.

The habitats used by juveniles were very different from subadults (Figure 6). The area used was also significantly different according to the result of the Wilcoxon rank-sum test (*p* < 0.01, Figure 7). Habitats (95% UD) used each year for each juvenile were at 17.48 ± 11.84 square kilometres, 206.99 ± 156.56 square kilometres for each subadult.

## 4. Discussion

### 4.1. Autumn and Spring Migration

Black-necked Cranes are altitude-driven migratory species, instead of latitude-driven [18]. This results in a difference in fundamental energy requirements depending on whether the migration is travelling towards higher or lower altitudes. In autumn, Black-necked Cranes in Tibet migrate from high breeding grounds to lower altitude wintering grounds where resources are more available. Conversely, spring migration requires Black-necked Cranes to travel to locations with higher altitudes, resulting in more energy consumption. In many species, spring migration duration is shorter due to the fact that early arrival on the breeding ground allows for higher breeding success [29,30]. Our study did not show a faster spring migration. Spring migration consisted of a longer migration distance and a less direct route, which is compensated by higher migration speed, resulting in a similar migration duration to autumn migration. The longer migration distance and the detours are likely due to the very fact that spring migration requires more energy in order to raise altitude. In autumn migration, the migration from high altitude to low altitude might allow for more effective flights. In spring, it needs to climb, and therefore is more challenging. Altitudinal changes might not be the only reason for longer and less direct spring migration routes. In Tibet, the wind direction is mostly southward [31], which aids in the southward autumn migration, and is more likely to hinder the northward spring migration. The less straight routes in spring migration might be a result of Black-necked Cranes avoiding difficult terrain to allow for quicker flight.

### 4.2. Juveniles and Subadults

**Figure 8:**
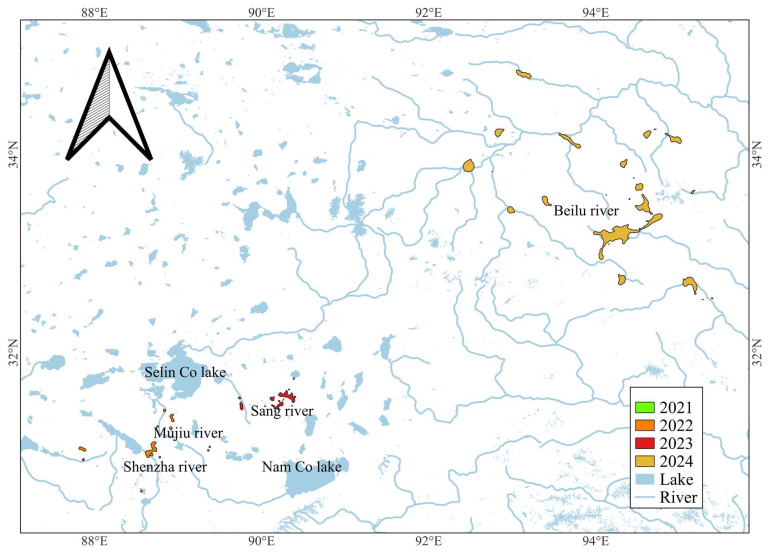
The summering/breeding habitat change of an individual (ID = 16) in four years.

In our study, juveniles used a very limited habitat area initially, and the area used grew as they became subadults. The individual ID = 16 in our study were tracked for four consecutive years, which showed low breeding/summering site fidelity. Such explorative behaviour in juveniles [32,33,34] could be a key driver of population expansion [24]. In the first year of ID = 16, its habitat use was a mere 11.02 square kilometres (2021), in the second year, this expanded to 221.18 square kilometres (2022); the third year, 254.59 square kilometres (2023); and the fourth year finally expanded to 2736 square kilometres (2024). In the first three years, it only stayed in its birthplace of the typical lake basin area in northern Tibet. In the fourth-year wintering period (2023 winter), it expanded to migrate from wintering grounds west of YZMR to wintering grounds east of YZMR. And only in the following spring did it migrate to SJYR in Qinghai for summering, and thus in 2024, its habitat area expanded dramatically. SJYR is the main breeding ground for another subpopulation of the Western population of the Black-necked Crane. This result shows that juveniles might be a key factor in facilitating inter-subpopulation communication (Figure 8).

### 4.3. Short-Distance Migration Facilitates Fast Population Recovery

The overall population trend of the Black-necked Crane has been partially increasing recently [35]. According to previous surveys [36], in 2022, the breeding population of Black-necked Cranes in Qinghai-Tibet Plateau was over 11,800; the breeding population in Yunnan–Guizhou Plateau (east population) was over 6000 individuals. Those numbers have increased compared to the numbers in 2012/2013 (over 6000 in Qinghai–Tibet Plateau; over 3900 in Yunnan–Guizhou Plateau) [37,38]. Compared to the eastern population (annual increase of 4.1%), the western population showed a faster growth rate (annual increase of 9.7%). We believe the main driver of the recent population growth in this species is the increase in suitable habitat. Climate change induced an overall trend of an increase in wetland area in Tibet [39,40,41] due to melting glaciers [42]. The expansion of wetlands provides more suitable habitats for the foraging, breeding, wintering and other activities throughout all the life stages of the Black-necked Crane.

The growth in the NT subpopulation is even higher. According to a breeding survey conducted during our study period, the annual growth rate of the NT subpopulation was an average of 12.17% (unpublished data by Yang), higher than the average of the western population. We deduce that the reason for this exceptionally high population growth rate of the NT subpopulation is its short migratory distance. According to previous studies [14,15,16,17,18,19,20], the migration distance of the NT subpopulation is shorter than other Black-necked Crane populations, except for Pumqu and Longbaotan/Shaluli (Table 2). The shortest migration distance in our study was only 163.97 km (Appendix A).

Migration is one of the life stages with the highest mortality in migratory species [43,44,45]. This is largely due to the high energetic demands of long-distance travel, which can exhaust individuals, especially juveniles or those in poor condition. In addition, migrants face numerous external threats during transit [46], such as extreme weather events, habitat loss at stopover sites, and anthropogenic pressures like collisions with power lines or hunting. Shorter migration distance means less mortality, fewer ecological barriers to cross and less dependence on stopover sites for survival. Because the migration distance is short, the refuel requirement during migration is less, and thus less dependency on high-quality stopover sites, and more resilience to adverse changes. With fewer requirements on stopover sites, there is a wider range of potential stopover sites to choose from, allowing more flexibility in migration routes, which explains the high inter-individual variability in our study. With shorter migration distances, the required fuel load for migration decreases and the cost for juveniles to learn the migration route is less. Those factors combined result in the fast growth of the population in the NT subpopulation. The link between migration distance and population growth can also be backed by the higher growth rate of the western population than the eastern, as the former has a shorter migration distance. The fast-growing subpopulations or populations like the NT subpopulation have the potential to be a source population for the entire species and aid in species recovery. Our study shows that the NT subpopulation has communication with other subpopulations, and a previous study has shown that there is communication between the Western population and other populations [18]. Therefore, the possibility of the high-growing population spilling over to other populations is high. It is of high conservation value to conserve those populations as well as those in fast decline.

### 4.4. Habitat Protection in the Heart of the Tibetan Plateau Will Be Key to the Conservation of Waterbirds Along the Central Asian Flyway

The Black-necked Crane, a first-class nationally protected species in China, serves as an indicator species for the Tibetan Plateau and the Central Asian flyway. Thanks to a series of government protection measures, including the establishment of national-level nature reserves, ecological restoration projects, and the enactment of laws like the Law of the People’s Republic of China on Ecological Protection of the Tibetan Plateau, significant strides have been made in the restoration and protection of Black-necked Crane habitats. As a result, the crane’s IUCN Red List status was downgraded from “Vulnerable” to “Near Threatened” in 2020 [12,47]. However, despite these conservation efforts, Black-necked Cranes continue to face significant threats from climate change and human activities. Climate change has led to dramatic shifts in the wetland ecosystems on the Tibetan Plateau, with rising temperatures, altered precipitation patterns, and increased frequency of extreme weather events potentially changing the hydrological conditions of wetlands [48]. For example, while glacial melting and increased rainfall have expanded some lake areas, permafrost degradation has led to the loss of shallow wetlands crucial for crane survival [49,50]. Additionally, human activities such as infrastructure development have further exacerbated habitat degradation. In the Lhasa River Basin, hydropower projects have altered river flow, reducing shallow water areas and severely limiting suitable habitats for the Black-necked Crane [51]. These environmental changes threaten not only Black-necked Cranes but also the broader wetland ecosystem that supports various migratory species.

As a critical node along the Central Asian flyway, the quality and extent of wetlands on the Tibetan Plateau directly influence the survival and migration success of a wide array of waterbird species [4]. However, the ongoing shrinkage of wetlands, degradation of vegetation, and increasing human activities have led to a significant decline in habitat quality and a reduction in the connectivity essential for waterbird migration [52]. This fragmentation of habitats has disrupted migration routes, making it more difficult for migratory species to find suitable breeding, foraging, and stopover sites. Moreover, climate-induced changes to the timing and availability of resources, such as food and water, have further complicated the migratory behaviour of Black-necked Cranes. High-quality stopover sites, which provide rest and energy replenishment during migration, are particularly critical to reducing survival risks related to energy expenditure and habitat competition [53]. The loss of these sites exacerbates the challenges cranes face during migration, leading to increased energy expenditure, higher risks of predation, and heightened competition for limited resources. In this context, Black-necked Cranes’ adoption of a short-distance migration strategy may reduce the risks and energetic costs associated with migration, while also supporting the stability and growth of their wintering population in Tibet.

Beyond these direct threats, broader environmental changes such as the shifting of species ranges due to climate change are also of concern [54,55]. As temperatures rise, many species, including those that share habitats with Black-necked Cranes, are moving to higher altitudes or latitudes, leading to increased competition for space and resources. The northward movement of species may alter the dynamics of migration corridors, creating new challenges for Black-necked Cranes as they navigate these shifting ecological landscapes. These environmental changes are forcing Black-necked Cranes to adapt to new conditions, potentially altering their migration, timing, and behaviour [56,57,58,59]. Therefore, in the context of future climate change, short-distance migration strategies may become even more crucial for the adaptive evolution of Black-necked Cranes. To support the long-term survival and resilience of this species, it is imperative to strengthen habitat connectivity along key short-distance migration corridors, such as the typical lake basin area in northern Tibet–Yarlung Tsangpo River corridor, and to consolidate a robust network of protected areas to maintain this “low-energy, high-efficiency” migration model. Effective conservation strategies must consider the broader impacts of environmental change, and we must act quickly to protect and restore habitats, ensuring the future sustainability of Black-necked Cranes and other migratory species that rely on the Tibetan Plateau’s wetlands.

### 4.5. Limitations and Future Directions

Our investigation into the NT subpopulation of Black-necked Cranes has revealed a distinctive “low-energy, high-efficiency” migratory strategy within a single, stable growth group. However, by concentrating on this one subpopulation and a four-year tracking window (2021–2024), variation may exist in other regional groups or across longer timeframes. We also focused on age-related shifts in habitat use, but did not explore how sex differences or individual migration history might influence movement. Moreover, although we framed our findings against the backdrop of a warming plateau and changing wetlands, we did not explicitly integrate environmental drivers, such as climate shifts or habitat degradation, into our movement analyses.

We believe future research can continue to build on these insights by: (1) expanding to additional subpopulations—especially those undertaking longer migrations—to test the generality of the NT pattern; (2) extending tracking efforts over a decade or more to capture interannual responses to climate and land-use shifts; and (3) examining individual-level factors such as sex, age, and prior migratory experience through larger samples and complementary methods, including drone surveys and citizen science data. Such integrative approaches will deepen our understanding of the mechanisms that shape crane migration and support more targeted, climate-adaptive conservation actions.

## 5. Conclusions

In this study, we used satellite tracking data from 16 Black-necked Cranes over 2021–2024 to reveal the migration strategies and habitat use patterns of the NT subpopulation—a little-known but rapidly growing group within the western population. We found that the NT subpopulation undertakes short-distance migrations within Tibet, with a mere mean distance of 284.21 km. These migrations were characterized by minimal reliance on stopover sites, particularly in autumn, and high inter-individual consistency in route selection. Statistically significant seasonal differences were detected: autumn migrations were shorter, more direct, slower, and at higher altitudes, while spring migrations were longer and more fragmented, likely due to the combined effects of topographical change and differences in individual life stages and reproductive status. We also found clear ontogenetic patterns in habitat use: juveniles initially occupied small and fragmented habitats, but their spatial range expanded significantly as they matured, with one individual eventually migrating to a different subpopulation’s summering/breeding area, highlighting the potential for dispersal and gene flow between subpopulations.

These findings collectively indicate that the NT subpopulation has adopted a unique “low-energy, high-efficiency” migration strategy tailored to the harsh conditions of the Tibetan Plateau. Such a strategy may reduce energetic costs and mortality risks, increase route flexibility, and allow earlier or more predictable arrival at breeding sites—advantages that likely contribute to the observed population increase in this region. Importantly, our results underscore the conservation value of short-distance migration corridors like the typical lake basin area in northern Tibet–Yarlung Tsangpo River system, which supports these movements. As environmental pressures mount due to climate change, habitat fragmentation, and infrastructure development, safeguarding the core breeding and wintering wetlands and maintaining connectivity along these short routes will be crucial. Our study not only provides new insights into black-necked crane migratory strategies but also provides a scientific basis for the protection of black-necked cranes.

## Figures and Tables

**Table 1:** Black-necked Crane satellite tracking information.

ID	Status at Capture	Tracking Period	Number of Locations
1	Juvenile	7 September 2023–28 July 2024	3365
2	Juvenile	7 September 2023–21 April 2024	5401
3	Juvenile	3 September 2023–12 September 2024	7887
4	Juvenile	7 September 2023–10 August 2024	7969
5	Juvenile	7 September 2023–14 August 2024	8132
6	Juvenile	31 August 2023–12 September 2024	8498
7	Juvenile	17 August 2021–12 September 2022	9508
8	Juvenile	31 August 2023–12 September 2024	7228
9	Juvenile	8 September 2023–12 September 2024	8354
10	Juvenile	1 September 2023–12 September 2024	8446
11	Juvenile	9 September 2023–12 September 2024	9315
12	Juvenile	9 September 2023–12 September 2024	9332
13	Adult	8 September 2023–12 September 2024	9426
14	Juvenile	31 August 2023–12 September 2024	9551
15	Juvenile	3 September 2023–12 September 2024	9653
16	Juvenile	18 August 2021–7 August 2024	25,717

**Table 2:** The migratory distance of subpopulations of the Black-necked Crane.

Breeding Sites-Subpopulation	Average Migration Distance (km)
Pumqu	112.62
Longbaotan/Shaluli	237.5
NT	284.21
The Southeastern Qiangtang	294.68
Manasarovar	654.33
Zoige	697.62
Pangong Tso	923.45
East Kunlun Mountains/Altyn Mountains	954.08
Qilian Mountains/Qinghai Lake	1338.49
Yanchiwan	1346.23

## Data Availability

The data presented in this study are available upon request from the corresponding author.

## Abbreviations

The following abbreviations are used in this manuscript:

IUCN	International Union for Conservation of Nature
NT	The subpopulation of the northern Tibetan lake basin
SLCR	Selin Co National Reserve
YZMR	Middle Reaches of the Yarlung Tsangpo River in Tibet Autonomous Region
HXR	Hoh Xil National Nature Reserve
SJYR	Sanjiangyuan National Nature Reserve
RKZ	Rikaze city
SN	Shannan city
LZ	Linzhou county
RR	Re River
GWQK	GeWangQueKang
DR	Dang River

## Appendix A

**Table A1:** List of Black-necked Crane autumn migration information.

Crane ID	Departure Date/Site	Arrive Date/Site	Migration Distance (km)
1	25 October 2023 Mujiu river	27 October 2023 Dazi county	212.71
2	19 October 2023 Shenzha river	20 October 2023 13:00 Qubuxiong county	222.09
4	19 October 2023 Shenzha river	20 October 2023 Bianxiong county	206.03
5	18 October 2023 Geren lake	19 October 2023 Zhaxigang county	235.05
7	20 October 2021 Shenzha river	22 October 2021 Xiang river	203.37
9	29 October 2023 Mujiu river	3 November 2023 Qubuxiong county	199.93
11	28 October 2023 Shenzha river	28 October 2023 Bianxiong county	212.71
13	29 October 2023 Mujiu river	3 November 2023 Qubuxiong county	241.32
16	22 October 2021 Shenzha river	22 October 2021 Bianxiong county	208.50
16	17 July 2022 Shenzha river	23 July 2022 Bianxiong county	206.54
15	29 October 2023 Bianqiong river	30 October 2023 Xiang river	231.51
10	26 October 2023 Coer lake	26 October 2023 Nianmu county	349.06
3	29 October 2023 Bianqiong river	30 October 2023 Xiang river	299.61
16	19 October 2023 Qiare river	20 October 2023 Nianmu county	293.95
14	5 November 2023 Qukeluo river	6 November 2023 Jiedexiu	276.21
6	5 November 2023 Qukeluo river	6 November 2023 Jiedexiu	274.75
8	20 October 2023 Beng lake	21 October 2023 Linzhou	163.97

**Table A2:** List of Black-necked Crane Spring Migration information.

Crane ID	Departure Date/Site	Arrive Date/Site	Migration Distance (km)
3	25 May 2024 Bianxiong county	26 May 2024 Xiagang river	337.41
4	3 April 2024 Bianxiong county	4 April 2024 Shenzha river	546.53
5	10 May 2024 Rongma county	14 May 2024 Duochajia river	612.43
7	6 May 2022 Bianxiong county	10 May 2022 Angcang river	644.87
9	22 March 2024 Aima county	22 March 2024 Mujiu river	241.43
10	14 May 2024 Nianmu county	14 May 2024 Geren lake	267.39
11	12 May 2024 Bianxiong county	16 May 2024 Bengna river	305.45
12	12 May 2024 Bianxiong county	14 May 2024 Nongren river	428.22
13	22 March 2024 Aima county	22 March 2024 Mujiu river	241.11
15	11 May 2024 Aima county	12 May 2024 Mujiu river	272.19
16	25 March 2022 Bianxiong county	25 March 2022 Shenzha river	209.59
6	22 April 2024 Jiedexiu	24 April 2024 Sang river	460.13
16	1 May 2023 Bianxiong county	3 May 2023 Sang river	459.62
6	22 April 2024 Jiedexiu	11 July 2024 Beilu river	867.59
16	13 April 2024 Jiedexiu	27 April 2024 Zhana river	633.35
